# Bis(4,4′-dimethyl-2,2′-bipyridine)oxidovanadium(IV) Sulfate Dehydrate: Potential Candidate for Controlling Lipid Metabolism?

**DOI:** 10.1155/2017/6950516

**Published:** 2017-04-26

**Authors:** Renata Francik, Jadwiga Kryczyk-Kozioł, Sławomir Francik, Ryszard Gryboś, Mirosław Krośniak

**Affiliations:** ^1^Department of Bioorganic Chemistry, Chair of Organic Chemistry, Jagiellonian University Medical College, Krakow, Poland; ^2^Department of Food Chemistry and Nutrition, Jagiellonian University Medical College, Krakow, Poland; ^3^Department of Mechanical Engineering and Agrophysics, Faculty of Production Engineering and Energetics, University of Agriculture in Krakow, Krakow, Poland; ^4^Faculty of Chemistry, Jagiellonian University, Krakow, Poland

## Abstract

Vanadium is a trace element mainly connected with regulation of insulin metabolism which is particularly important in diabetes. In recent years, organic complexes of vanadium seem to be more interesting than inorganic salts. Nevertheless, the effect of vanadium on lipid metabolism is still a problematic issue; therefore, the main purpose of this study was to investigate the effect of 3 organic complexes of vanadium such as sodium (2,2′-bipyridine)oxidobisperoxovanadate(V) octahydrate, bis(2,2′-bipyridine)oxidovanadium(IV) sulfate dehydrate, and bis(4,4′-dimethyl-2,2′-bipyridine)oxidovanadium(IV) sulfate dihydrate in conjunction with high-fat as well as control diet in nondiabetes model on the following lipid parameters: total cholesterol, triglycerides, and high density lipoprotein as well as activity of paraoxonase 1. All of these parameters were determined in plasma of Wistar rats. The most significant effect was observed in case of bis(4,4′-dimethyl-2,2′ bipyridine)oxidovanadium(IV) sulfate dehydrate in rats fed with high-fat diet. Based on our research, bis(4,4′-dimethyl-2,2′-bipyridine)oxidovanadium(IV) sulfate dihydrate should be the aim of further research and perhaps it will be an important factor in the regulation of lipid metabolism.

## 1. Introduction

Vanadium is a trace element on which a lot of attention was paid in context of diabetes. Initially, vanadium compounds were supposed to be insulin mimetic; however, now this element is mainly considered as a mean of increasing sensitivity of hepatocytes and myocytes on the effect of this hormone. Regulation by vanadium among other activities of the protein tyrosine phosphatase-1B (PTP-1B) is particularly important [1]. Probably the excessive activity of protein tyrosine phosphatase can affect insulin resistance [2]. The effect of vanadium compounds on glucose transport involving protein GLUT-4 was indicated too [3, 4]. What is more, this element activates also enzymes involved in glycolysis and glycogenesis as well as lipid metabolism [5].

Nowadays, the effect of vanadium on carbohydrate metabolism is getting better known, while the effect on lipid metabolism is still a problematic issue. Study indicating dyslipidemic properties of organic complexes of vanadium was conducted in diabetes model [6]; nevertheless, there is still lack of research evaluating these properties in nondiabetes model. Disturbance of lipid metabolism is a basic factor in development of cardiovascular diseases, which are currently one of the main reasons for mortality [7, 8]. Statins are common drugs used in treatment of such patients. However, there is an urgent need of search for alternative forms of treatment for patients with contraindications of statins. Therefore, the main purpose of this study was to investigate the effect of 3 organic vanadium complexes on selected lipid parameters in rats fed with a high-fat diet.

Paraoxonase 1 (PON1) is an enzyme involved in lipid metabolism, for example, by limiting HDL and LDL oxidation. Moreover, this enzyme is also known for anti-inflammatory properties [9]. Meta-analyses indicated a correlation between risk of cardiovascular disease and PON1 activity among people independent of age or ethnicity [10, 11]. On the other hand, inadequate diet can contribute to reduction level of expression of that enzyme [12]. Taking into account a protective role of PON1 in cardiovascular diseases, we found it also necessary to evaluate the influence of these vanadium complexes on this parameter. Because this enzyme is calcium-dependent, we analyzed this element as well.

## 2. Materials and Methods

### 2.1. Reagents

The reagents were purchased from Sigma Aldrich Chemical Company (Steinheim, Germany) and Avantor Performance Materials Poland S.A.

### 2.2. Synthesis of Vanadium Complexes

In this study, the following complexes were used: sodium (2,2′-bipyridine)oxidobisperoxidovanadate(V) octahydrate, Na[VO(O_2_)_2_(2,2′-bpy)]·8H_2_O, marked as V (453.9 g/mol); bis(2,2′-bipyridine)oxidovanadium(IV) sulfate dihydrate, [VO(SO_4_)(2,2′-bpy)]·2H_2_O, marked as B (511.21 g/mol); and bis(4,4′-dimethyl-2,2′-bipyridine)oxidovanadium(IV) sulfate dihydrate, [VO(4,4′-Me-2,2′-bpy)_2_]SO_4_·2H_2_O marked as Bm (567.21 g/mol). Synthesis of the first compound V was described [13]. In turn, synthesis of compound B was described [14]. In case of Bm compound, process of synthesis was similar to B complex except molar ratio of ligand to vanadium which was 2 : 1. The purity of all analyzed complexes was confirmed by microanalysis and IR spectroscopy.

### 2.3. Animals

In the experiment, male Wistar rats aged 3 months and weighing 250 ± 15 g were divided into 8 groups of 6 animals. During the time of experiment (5 weeks), each group of animals was fed with different diet: group CN, standard diet (starch—62%, casein—20%, oil—5.0%, calcium carbonate—2.8%, Ca_3_(PO_4_)_2_—2.9%, lecithin—1.0%, NaCl—0.3%, cellulose—4.7%, minerals and vitamins mix.—1.0%, MgO—0.07%, and K_2_SO_4_—0.23%); group CV, standard diet + vanadium complex V; group CB, standard diet + vanadium complex B; group CBm, standard diet + vanadium complex Bm; group AN, high fatty diet (starch—32%, casein—20%, oil—5.0%, lard—30%, calcium carbonate—2.8%, Ca_3_(PO_4_)_2_—2.9%, lecithin—1.0%, NaCl—0.3%, cellulose—4.7%, minerals and vitamins mix.—1.0%, MgO—0.07%, and K_2_SO_4_—0.23%); group AV, high fatty diet + vanadium complex V; group AB, high fatty diet + vanadium complex B; group ABm, high fatty diet + vanadium complex Bm. In all vanadium treated groups, tested complexes were administered by gavage once a day during 5 weeks in the dose of 20 mg/kg body mass.

All animals had free access to feed and water and were kept in a room with a constant temperature of 23°C and 50–60% humidity with a 12-hour day/night cycle. After 5 weeks, the animals were anesthetized. This experience was conducted with an approval of I Local Ethics Committee for Animal Experiments of Jagiellonian University in Cracow, number 80/2009 17.09.2009.

### 2.4. Blood Preparation

Blood samples were taken from aorta into heparinized tubes and then centrifuged (at 2500 ×g for 15 minutes at 4°C) to obtain plasma which was kept frozen (at −80°C) until further analyses.

### 2.5. Biochemical Parameters

Biochemical analysis was done using standard biochemical analyzer Alizé BioMerieux with standard kits (Ca—61041, TCHOL—61218, TG—61236, HDL—61001 and 61002) from BioMerieux. Thus, the obtained results were compared with Control Serum 1, ODC0003, and Control Serum 2, ODC0004 (OLYMPUS).

### 2.6. Paraoxonase 1 Activity (PON1) in Plasma

Paraoxonase 1 (PON1) activity was determined by modified Eckerson method [15]. A mixture of 0.25 M Tris buffer (pH 8) and 0.1 M paraoxon in a volume ratio of 19 : 1 (v/v) was added to samples. Then, the absorbance was measured at 412 nm during 2 minutes. PON1 activity was estimated based on changes in concentration of substrate.

### 2.7. Statistical Analysis

We used two-way analysis of variance (ANOVA) in order to verify if the examined factors (explanatory variables) influence the observed (dependent) variables [16]. Before this, we had checked if the main assumptions concerning normal distribution of dependent variable within groups as well as homogeneity of variance were met [17]. Normal distribution was verified by Shapiro-Wilk test while homogeneity of variance was verified by Levene's test. In turn, Box-Cox transformation was used for variables which did not meet ANOVA assumptions [17, 18].

Analysis of variance was conducted for each of the following dependent variables: Ca [mmol/l], TCHOL [mmol/l], TG [mmol/l], HDL [mg/dL], and PON1 [U/mg protein]. Intragroup factors were the type of diet (X1-diet) and type of supplement (X2-supplement). Type of diet (X1-diet) had 2 levels (C and A); in turn, type of supplement (X2-supplement) had 4 levels (N, B, V, and Bm).

Tukey's test was used to indicate homogeneous groups (marked by identical letters). All analyses were conducted at significance level *p* = 0.05. Obtained data were statistically analyzed using the STATISTICA (StatSoft, Inc. (2011) version 10, http://www.statsoft.com.).

Based on Levene's test, 3 variables such as TCHOL [mmol/l], TG [mmol/l], and PON1 [U/mg protein] did not meet assumption of homogeneity of variance. Therefore, for them, Box-Cox transformation was made after which variables met the assumption concerning normal distribution (Shapiro-Wilk test) and homogeneity of variance (Levene's test) at significance level *p* = 0.05. Then, two-way analysis of variance (ANOVA) was used.

## 3. Results

The results are summarized in Table 1. All parameters were analyzed in plasma of rats. Bm vanadium complex in control diet (CBm) statistically increased (*p* < 0.05) concentration of calcium compared to control diet (CN). In case of V vanadium complex in control diet (CV), activity of PON 1 significantly decreased (*p* < 0.05) compared to control diet (CN) and B vanadium compounds with control diet (CB). In other tested parameters like TCHOL, TG, and HDL, we did not note significant changes.

In turn, V vanadium complex with high-fat diet (AV) statistically increased (*p* < 0.05) concentration of calcium compared to other vanadium complexes as well as high-fat diet (AB, ABm, and A). B as well as Bm complexes with high-fat diet (AB, ABm) statistically increased (*p* < 0.05) level of TCHOL compared to high-fat diet (A). Opposite dependence for these compounds was observed for activity of PON1. Rats fed with high-fat diet in AB and ABm groups had higher level of HDL in plasma than AN or AV groups. V as well as B complexes with high-fat diet (AV, AB) statistically increased (*p* < 0.05) the level of TG compared to AN and ABm group.

## 4. Discussion

The role of vanadium in the human body has not been fully understood yet. This trace element is still subject of numerous biochemical studies to understand its significance to human health. Recently, vanadium is considered essential to life. Previous studies were primarily related to its antidiabetic properties [19, 20]. In animal models of diabetes types 1 and 2, research was conducted mostly into vanadium complexes with organic ligands, for example, bis(maltolato)oxidovanadium(IV), bis(picolinato)oxidovanadium, vanadyl acetylacetone, and vanadyl 3-ethylpentane-2,4-dione (Vet) [21, 22]. Organic complexes of vanadium seem to have stronger effects and gain an advantage over inorganic salts as potential antidiabetic agents. What is more, such complexes have not shown adverse effects on gastrointestinal tract. Therefore, in this study, we used organic complexes of vanadium(IV) (B and Bm complexes) and vanadium(V) (V complex), which contain a bipyridine as an organic ligand. In addition, bipyridine in Bm complex was substituted at position 4 by methyl group. The presence of additional group at heterocyclic ring of this complex contributed to increasing lipophilicity of it which may have importance for enhanced bioavailability. In our study, we focused on assessing the organic vanadium complex on lipid metabolism under conditions of high-fat diet in nondiabetes animal model due to the lack of such research. This was the main reason for our experiment.

B and Bm complexes with high-fat diet statistically increased (*p* < 0.05) the level of TCHOL. One of the potential explanations for the increased value of this parameter would be the fact that those compounds also significantly elevated (*p* < 0.05) the level of HDL. However, an important limitation of our study was not to determine the level of LDL. In rats fed with control diet, we did not note any changes induced by one of vanadium complexes. Similarly, Majithiya et al. observed that vanadium complex like bis[curcumino]oxidovanadium adjusted values of lipid parameters (decreased level of TCHOL and TG) in streptozotocin induced diabetic rats, without any impact on control nondiabetes rats [23]. Pillaia et al. also noted no changes in the level of lipids parameters in nondiabetic rats receiving vanadium-3-hydroxy flavone complex. In turn, in diabetic rats, the same compound showed antidyslipidemic properties [6].

In our study, B and Bm complexes significantly increased (*p* < 0.05) the level of HDL in combination with a high-fat diet. Alike results were obtained among people exposed to vanadium compounds [24] as well as rabbits fed with a high-cholesterol diet supplemented with vanadium (as sodium metavanadate) [25]. Additionally, Bm complex in high-fat diet as the only one of used organic complexes of vanadium did not significantly increase the concentration of TG. These results encourage us to further research this particular compound. Nevertheless, it is possible too that hypolipidemic effect of different vanadium complexes is strong in the presence of diabetes, contrary to high supply of saturated fatty acids, but without carbohydrate disorders.

The consequence of oxidative stress is lowering of PON1 activity as observed in the course of coronary artery disease [26]. Therefore, the attempt to regulate the activity of this enzyme may seem particularly significant in treatment of this disease. However, in our studies, B and Bm complexes of vanadium in conjunction with a high-fat diet caused a statistical decrease (*p* < 0.05) in the activity of PON1. Further V complex administered with control diet affected PON1 in a similar way. In turn, Tas et al. noted that vanadyl sulfate with standard diet did not influence these parameters in healthy rats [26]. The lack of significant difference in PON1 activity between AN and CN group seems to be quite puzzling. Nevertheless, in the studies on the effect of fatty diet on PON1, there are discrepancies too; for example, Thomàs-Moyà et al. noted a decrease in the activity of PON1 in animals fed with a high-fat as well as hypercholesterolemic diets [27]. Kudchodkar et al. observed that diet rich in fish oil significantly reduces while trioleate increases the activity of it. In turn, in tripalmitate fed rats, no significant change of activity of this parameter was observed compared to control diet [28]. In a study on people, Calabresi et al. have noted a significant increase in the activity of PON1 in patients with hyperlipidemia who received *ω*-3 polyunsaturated fatty acid for 8 weeks compared to the placebo group [29]. Therefore, the results in these studies indicate how the type of fatty acids is an important factor in regulating the activity of PON1. What is more, the time of our experiment perhaps was too short to observe change in this parameter between AN and CN group. Nevertheless, the quite unexpected results obtained by Kleemola et al. are also worth noting where activity of PON negatively correlated with consumption of foods rich in antioxidants [30]. This is another proof of the complexity of this issue and the necessity to further research to understand the interaction between PON1 activity and specific chemical or food component.

## 5. Conclusions

In the present paper, we have demonstrated that bis(4,4′-dimethyl-2,2′-bipyridine)oxidovanadium(IV) sulfate dehydrate (Bm) compound is an interesting object to further study as a potential candidate for controlling lipid metabolism in condition of high supply of fats, although molecular studies on this particular compound are necessary to find out potential intracellular mechanisms of diminish side effect of fat diet.

## Figures and Tables

**Table 1:** Biochemical parameters. Data are presented as means from independent measurements ± standard deviation (SD).

X1-diet	X2-supplement	Name group	Ca [mmol/l]	TCHOL [mmol/l]	TG [mmol/l]	HDL [mg/dL]	PON1 [U/mg protein]
C	N	CN	3.13 ± 0.27^a^	1.99 ± 0.48^a^	1.28 ± 0.14^bc^	33.11 ± 6.60^ab^	366 ± 98^bc^
C	V	CV	3.2 ± 0.15^ab^	1.98 ± 0.36^a^	1.21 ± 0.09^c^	39.65 ± 6.58^ab^	207 ± 43^a^
C	B	CB	3.23 ± 0.17^ab^	1.56 ± 0.41^a^	1.41 ± 0.30^c^	39.87 ± 8.40^ab^	362 ± 97^bc^
C	Bm	CBm	3.48 ± 0.33^b^	1.72 ± 0.36^a^	1.56 ± 0.44^c^	41.49 ± 7.40^a^	311 ± 52^abc^
A	N	AN	3.03 ± 0.11^a^	1.76 ± 0.04^a^	0.63 ± 0.13^a^	25.68 ± 9.01^b^	386 ± 23^c^
A	V	AV	3.36 ± 0.19^b^	2.15 ± 0.30^ab^	1.13 ± 0.12^bc^	35.34 ± 2.60^b^	313 ± 56^abc^
A	B	AB	3.04 ± 0.21^a^	2.17 ± 0.53^b^	1.11 ± 0.24^bc^	41.99 ± 8.00^a^	260 ± 54^ab^
A	Bm	ABm	2.97 ± 0.33^a^	2.80 ± 0.36^b^	0.84 ± 0.17^a^	41.89 ± 8.10^a^	202 ± 55^a^

CN: control diet without additives; CV: V vanadium compounds with control diet; CB: B vanadium compounds with control diet; CBm: Bm vanadium compounds with control diet; AN: high-fat diet without additives; AV: V vanadium compounds with high-fat diet; AB: B vanadium compounds with high-fat diet; ABm: Bm vanadium compounds with high-fat diet.

Ca: calcium; TCHOL: total cholesterol; TG: triglycerides; HDL: high-density lipoprotein, PON1: activity of paraoxonase 1.

Means in the same columns followed by different letters (a, b, c) are significantly different at *p* < 0.05 according to Tukey's test.

## Abbreviations

Compound V: Sodium (2,2′-bipyridine)oxidobisperoxidovanadate(V) octahydrate, Na[VO(O_2_)_2_(2,2′-bpy)]·8H_2_O
Compound B: Bis(2,2′-bipyridine)oxidovanadium(IV) sulfate dihydrate, [VO(SO_4_)(2,2′-bpy)]·2H_2_O
Compound Bm: Bis(4,4′-dimethyl-2,2′-bipyridine)oxidovanadium(IV) sulfate dihydrate, [VO(4,4′-Me-2,2′-bpy)_2_]SO_4_·2H_2_O
GLUT-4: Glucose transporter type 4
TCHOL: Total cholesterol
TG: Triglycerides
HDL: High density lipoprotein
PON1: Paraoxonase 1.
